# Function‐Specific Localization in the Supplementary Motor Area: A Potential Effective Target for Tourette Syndrome

**DOI:** 10.1111/cns.70280

**Published:** 2025-02-21

**Authors:** Jue Wang, Juan Yue, Ye Wang, Xiao‐Long Li, Xin‐Ping Deng, Yu‐Ting Lou, Liu‐Yan Gao, Xiao‐Quan Chen, Qun‐Yan Su, Yu‐Feng Zang, Jian‐Hua Feng

**Affiliations:** ^1^ Institute of Sports Medicine and Health Chengdu Sport University Chengdu China; ^2^ TMS Center Hangzhou Normal University Affiliated Deqing Hospital Huzhou China; ^3^ Department of Pediatrics, the Second Affiliated Hospital Zhejiang University School of Medicine Hangzhou China; ^4^ Center for Cognition and Brain Disorders The Affiliated Hospital of Hangzhou Normal University Hangzhou China; ^5^ Department of Pediatrics Taizhou Woman and Children's Hospital Taizhou China

**Keywords:** functional connectivity, function‐specific target, left supplementary motor area, Tourette's syndrome

## Abstract

**Aims:**

Repetitive transcranial magnetic stimulation (rTMS) targeting the supplementary motor area (SMA) may treat Tourette's syndrome (TS) by modulating the function of the globus pallidus internus (GPi) via the cortico‐striato‐thalamo‐cortical circuit.

**Methods:**

We conducted a randomized longitudinal study to examine circuit functionality and clinical efficacy. The GPi was identified as an “effective region” for TS treatment. Using functional MRI, individualized targets within the SMA were identified. Function‐specific targets [left SMA (*n* = 19), right SMA (*n* = 16)] were compared with conventional scalp‐localized SMA targets (*n* = 19). Age‐ and gender‐matched typical developmental children (TDC) served as controls (*n* = 48). TS patients received 50 Hz continuous theta burst stimulation (cTBS) at 70% RMT over five consecutive days (1800 pulses/day). Clinical efficacy was assessed using the Yale Global Tic Severity Scale (YGTSS) at one and two weeks post‐cTBS. Functional connectivity (FC) analyses of the GPi evaluated the impact on brain function.

**Results:**

There was an approximately 3 cm Y‐axis distance between the function‐specific and conventional targets. TS patients exhibited significantly reduced GPi‐base FC in bilateral motor areas at baseline compared to TDC. Following cTBS, 4 out of 19 patients in the left SMA group achieved a ≥ 30% reduction in YGTSS scores. cTBS modulated brain function in the left inferior orbital frontal cortex and right Lingual/cerebellum, primarily influenced by the right SMA target, whereas the conventional target showed no effect on YGTSS scores. Changes in GPi‐target FC were significantly correlated with reduction in YGTSS total scores (*r* = 0.638, *p* = 0.026).

**Conclusion:**

These findings suggest that function‐specific SMA targets may yield more pronounced modulatory effects, with the left SMA target achieving “Effectiveness” after just one week of cTBS. Combining function‐specific SMA‐targeted cTBS with standard treatment shows promise in accelerating clinical efficacy for TS treatment, warranting further investigation.

## Graphical abstract

Combining function‐specific SMA‐targeted cTBS with standard treatment may be more effective than conventional SMA targeting in accelerating the onset of clinical efficacy in TS treatment.

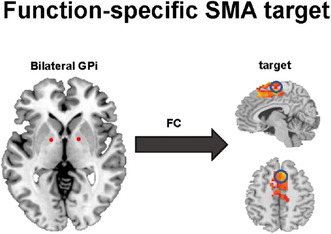

## 1 Introduction

Transcranial magnetic stimulation (TMS) is typically utilized to study the neural networks of central motor pathways and to modulate brain function for therapy in neurological disorders [1], including tic disorders [2]. Tourette's syndrome (TS) is characterized by chronic motor and vocal tics, with persistent motor tics significantly impacting children's learning and quality of life. Consequently, targeting the motor cortex became a natural focal point for stimulation.

Application of 6 months of 1‐Hz rTMS treatment to the bilateral supplementary motor areas (SMA) can achieve a reduction in tic severity [3, 4], whereas 1‐Hz rTMS to the left primary or premotor cortex was reported to have no impact on tic frequency or severity [5]. A recent study using individualized functional magnetic resonance imaging (fMRI)‐guided continuous theta burst stimulation (cTBS) showed a promising modulatory effect. While no difference in tic reduction was observed between active and sham groups at 7 days, the active groups exhibited a significant decrease in task activation in both SMA and bilateral M1 after only 2 days of cTBS treatment [6]. Recent studies have shown that rapid therapeutic effects can be achieved by applying high‐density stimulation over short durations [7, 8, 9, 10]. Animal studies have demonstrated that the SMA primarily receives inputs originating from the GPi [11]. Thus, the SMA may be a better rTMS target than M1 for the TS treatment [5, 6, 12].

The conventional scalp‐based targeting method for the SMA involves measuring the distance from the nasion to the inion. The target is located at a point 15% of this distance anterior to the Cz point in the International 10–20 EEG system. This position lies along the brain's midline. If the measurement precision is sufficiently high, the stimulation might precisely target the cerebrospinal fluid (CSF), resulting in no therapeutic effect. However, due to the inherent asymmetry of the human brain, some patients may receive stimulation to either the left or right SMA, leading to inconsistent therapeutic outcomes. Given the SMA's extensive spatial distribution, it is crucial to precisely define its specific location. Studies demonstrating the success of fMRI‐guided rTMS in treating depression [13] have underscored the technique's potential. The most promising approach for rTMS involves functional connectivity (FC)‐guided targeting. Previous research has indicated that targets located near areas exhibiting maximal FC anticorrelation with the subgenual anterior cingulate cortex (SGC) within the dorsal lateral prefrontal cortex (DLPFC) tend to achieve better clinical efficacy compared to more distant targets [14, 15]. Individualized SGC‐FC‐guided TMS has been successfully applied in depression treatment [10], suggesting that rTMS targeting the DLPFC may indirectly influence the “effective region” within the SGC. The globus pallidus internus (GPi), situated within the cortico‐striato‐thalamo‐cortical (CSTC) circuit, represents one of the most frequently targeted regions for surgery‐based deep brain stimulation (DBS) in treating TS [16, 17, 18]. Therefore, we consider it a potentially “effective region” for rTMS treatment.

Evidence supporting the validity of individualized FC‐guided rTMS comes from studies of memory, a function widely associated with the hippocampus [19, 20, 21]. In other words, rTMS may alter hippocampal functioning. The proximity of the target to the voxel with the peak FC value is crucial, as targets closer to this peak usually yield better clinical outcomes compared to those further away. This suggests that brain networks identified by FC could potentially serve as a “bridge” to transmit rTMS effects to effective regions, such as the GPi.

**FIGURE 1:**
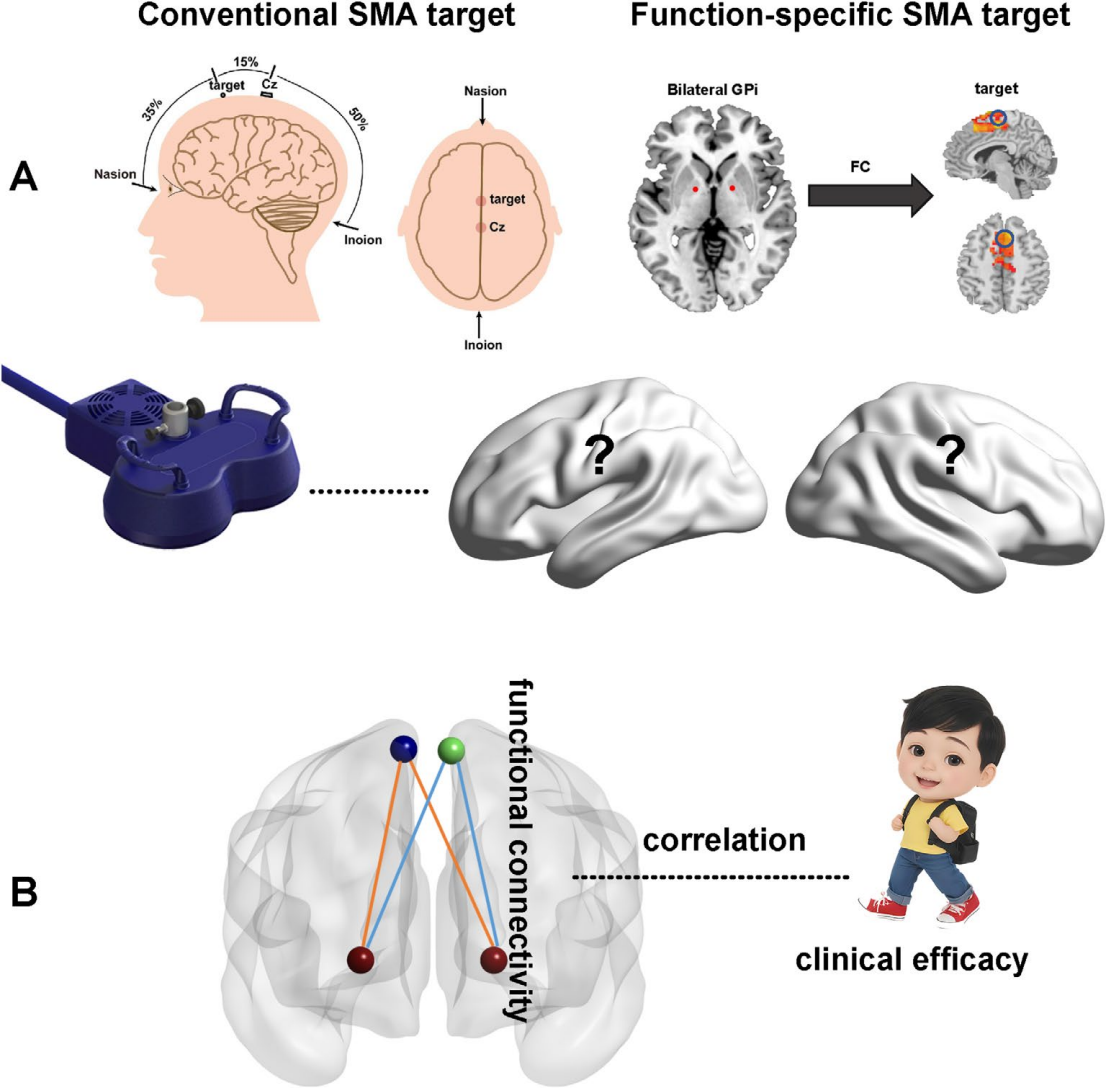
Visualizing the hypothetical model workflow. (A) The impact of the left or right target in SMA on treatment outcomes varies, with the conventional target located along the midline of the brain. (B) Functional alterations of GPi‐target is associated with clinical efficacy. GPi: Globus pallidus internus; SMA: Supplementary motor area.

An FC‐guided target not only provides individualized and precise localization but also establishes a connection with the effective target (GPi) of DBS. In this study, we defined an individualized GPi‐based peak FC coordinate in the SMA as the stimulation target. Additionally, a conventional scalp‐based SMA target was defined for comparison purposes. This allowed us to explore whether cTBS can normalize brain function and whether a function‐specific target offers advantages over a conventional target. Furthermore, we assessed whether cTBS could accelerate the time to achieve clinical efficacy when used in conjunction with standard treatment and investigated potential variations in treatment outcomes between left and right SMA targets (with the conventional target positioned along the midline of the brain) (Figure 1A). Finally, we examined whether functional alterations of the GPi‐SMA are associated with clinical efficacy (Figure 1B).

## 2 Materials and Methods

### 2.1 Participants

This study received approval from the Ethics Committee of the Center for Cognition and Brain Disorders (CCBD) at Hangzhou Normal University (HZNU) and was registered on www.clinicaltrials.gov (NCT04128397 for “An Exploratory Study of Continuous Theta Burst Stimulation (cTBS) Based on fMRI in the Treatment of Tic Disorder” and NCT02144467 for “The Establishment of Large‐sample Database of ‘Multiple‐MRI/Gene/Cognition’”). Patient recruitment commenced on June 29, 2018. Participants provided their consent in accordance with the Declaration of Helsinki, and both participants and their parents provided written informed consent before joining the study. Informed consent was obtained by the neurologists (Wang Y., Lou Y.T., Chen X.Q.).

The current study employs a multi‐arm parallel‐group randomized design. Initially, ninety‐five patients with TS from the Department of Pediatrics, Second Affiliated Hospital, Zhejiang University School of Medicine, underwent screening for eligibility. Patients were randomized in a 1:1 ratio to either the function‐specific target group or the conventional target group based on standard treatment protocols. Sixty‐one patients were enrolled in the SMA target arm. Despite initially planning to recruit 64 patients per group as calculated by G*Power, the study was prematurely concluded due to the influenza and subsequent COVID‐19 pandemic. The actual completion date of the study was December 31, 2019. Therefore, this report focuses specifically on the SMA target arm, where recruitment was nearly completed according to the original plan.

**FIGURE 2:**
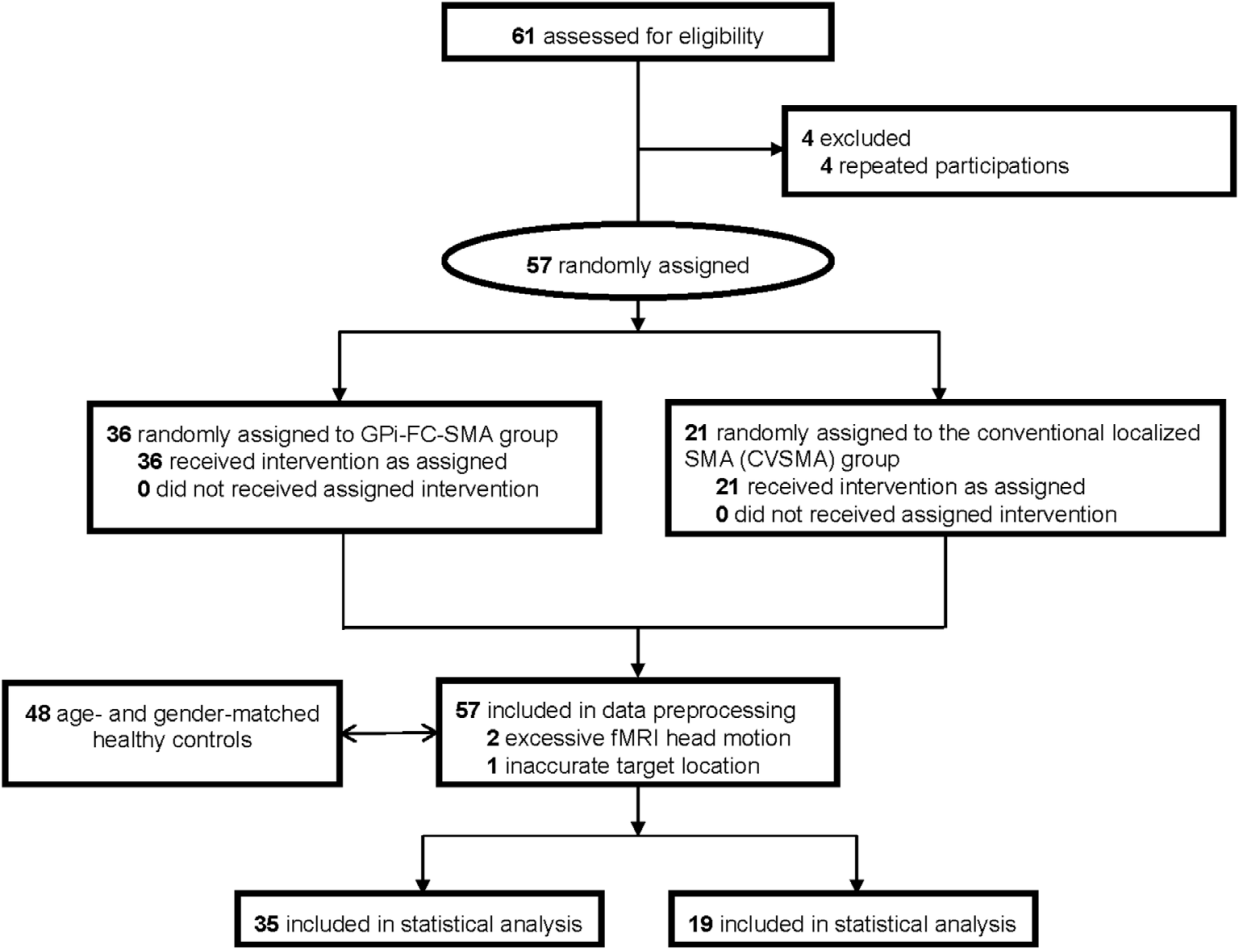
Trial profile.

All TS patients met the criteria outlined in the Diagnostic and Statistical Manual of Mental Disorders, 5th edition, text revision (DSM‐V‐TR). Four patients were excluded for participating twice, eight due to head motion exceeding 3 mm of translation or 3° of rotation in any direction, and one for inaccurate target location. Ultimately, 54 TS patients (aged 6.5 to 18 years, mean age 11.73 ± 2.87 years, including 10 girls) underwent statistical analysis, with 35 in the GPi‐SMA group and 19 in the conventional scalp localized target of the SMA (CVSMA) group (Figure 2).

A cohort of 48 typical developmental children (TDC), matched for age and gender, was selected from the comprehensive CCBD dataset. These controls exhibited no neurological or psychiatric disorders, nor any observable abnormalities in brain structure. Patients with TS showed no neurological conditions other than tics, and psychiatric comorbidities were limited to attention‐deficit hyperactivity disorder and obsessive‐compulsive disorder. No structural abnormalities were observed upon visual inspection of structural imaging.

### 2.2 Randomization

Randomization was conducted using a computer‐based random number table procedure. Numbered slips of paper were then placed in opaque, sealed envelopes by Wang J. Assignment of patients to either the conventional target or function‐specific target groups was performed by staff members not involved in treatment or clinical evaluation (Su Q.Y.). Both patients and neurologists responsible for enrollment and clinical evaluation (Wang Y., Lou Y.T., Chen X.Q.) were blinded to the treatment targets. Additionally, therapists (Yue J., Li X.L., Deng X.P.) administering the stimulation were also blinded to clinical evaluations. All information relating to group assignments remained concealed until the conclusion of the experiment. In cases where blinding was compromised, patients would be withdrawn from the RCT, while the intervention would continue.

### 2.3 Data Acquisition

Wang Y and Yue J were responsible for all the data entry and storage in this study. Zang Y.F. and Feng J.H. were temporarily responsible for data monitoring until the establishment of the Data Monitoring Committee. All personal information of patients and participants was collected, shared, and maintained in electronic and paper documents by the hospital. Electronic documents were not shared via the internet before, during, or after the trial.

### 2.4 Tic Severity Measures

**FIGURE 3:**
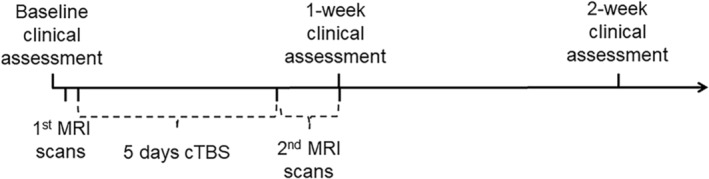
Experimental flowchart.

The primary outcome is clinical efficacy, with the secondary outcome being alteration in brain function. The Yale Global Tic Severity Scale (YGTSS) was used to assess current tic severity. All clinical evaluations were performed by experienced pediatric neurologists (Dr. Wang Y., Lou Y.T., Chen X.Q.) both before the MRI scans and one week after stimulation initiation. Assessments were also conducted for a subset of patients two weeks post‐stimulation onset. The YGTSS was employed to evaluate symptoms in the week following treatment. For detailed experimental procedures, please refer to Figure 3 (Figure 3). We ensured that at least one clinical assessment was conducted after completing 5 days of cTBS. Demographic information and clinical assessment details can be found in Table 1 and Table 2.

**TABLE 1:** Demographical, clinical, and neurophysiological information at baseline.

Characteristic demographics	GPi‐FC‐SMA group (*n* = 35)	CVSMA group (*n* = 19)	Sig. (*P* value)	TDC (*n* = 48)	Sig. (*P* value) compared with all TS patients
Age (mean ± SD)	11.74 ± 3.06	11.68 ± 2.45	0.949	11.21 ± 1.65	0.277
Male gender (*n*, %)	29, 83%	15, 79%	0.887 (Chi‐Square Test)	33, 69%	0.19
Medication Status					
Antipsychotic	17 (49%)	7 (37%)	0.440 (Fisher's Exact Test)		
Alpha‐2 agonist	1 (3%)	0 (0%)			
Chinese traditional medicine	1 (3%)	2 (11%)			
Any other tic medication	3 (9%)	3 (16%)			
Co‐Occurring Psychiatric Diagnoses					
ADHD	9 (26%)	10 (53%)	0.840 (Fisher's Exact Test)		
OCD	2 (6%)	3 (16%)			
SAD	1 (3%)	0 (0%)			
ODD	0 (0%)	1 (5%)			
One or more co‐occurring psychiatric diagnosis	1 (3%)	3 (16%)			

Abbreviations: CVSMA, conventional localized; FC, functional connectivity; GPi, globus pallidus internus; OCD, obsessive‐compulsive disorder; ODD, oppositional defiant disorder. SAD, social anxiety disorder; SMA, supplementary motor area; SMA; ADHD, attention deficit hyperactivity disorder; TDC, typical developmental children; TS, Tourette's syndrome.

**TABLE 2:** Clinical efficacy measures (baseline, 1‐week, and 2‐week after the first cTBS treatment) for TS patients.

Rating score	Baseline^a^ (mean ± SD, Median)	1‐week^a^ (mean ± SD, Median)	*P* value (Friedman test)	2‐week^b^ (mean ± SD, Median)	*P* value (Friedman test)
YGTSS‐M	14.83 ± 5.96, 17	13.83 ± 5.87, 15	< 0.0001	11.92 ± 5.58, 14	0.032
YGTSS‐P	11.61 ± 7.70, 14	10.50 ± 7.17, 12	< 0.0001	8.08 ± 5.63, 9	0.2
YGTSS‐I	20.19 ± 11.57, 20	19.35 ± 11.58, 20	0.059	5.83 ± 6.69, 5	0.002
YGTSS‐Total	46.63 ± 17.27, 47	43.69 ± 17.22, 42	< 0.0001	25.83 ± 10.74, 25	0.005^c^

Abbreviations: TS, Tourette's syndrome; YGTSS, Yale Global Tic Severity Scale; YGTSS‐I, Yale Global Tic Severity Scale Impairment; YGTSS‐M, Yale Global Tic Severity Scale Motor; YGTSS‐P, Yale Global Tic Severity Scale Phonic.

^a^ *N* = 54.

^b^ *N* = 12.

^c^ Bonferroni correction of Related‐samples Friedman's two‐way analysis of variance by ranks test.

### 2.5 MRI Acquisition Procedure

MRI scans were conducted both prior to the initiation of stimulation and one week after the commencement of stimulation. MRI data were acquired on a 3‐T scanner (MR‐750, GE Medical Systems, Milwaukee, WI) at the Center for Cognition and Brain Disorders of Hangzhou Normal University. Comfortable straps and foam pads were placed to minimize head motion. A high‐resolution T1‐weighted anatomical image was first acquired using a 3D spoiled gradient echo sequence with the following parameters: sagittal slices; slice number, 176; matrix size, 256 × 256; field of view (FOV), 256 × 256 mm; repetition time (TR)/echo time (TE), 8.1/3.1 ms; FA, 8°; and thickness/gap, 1/0 mm (isotropic voxel size, 1 × 1 × 1 mm). The fMRI images were then acquired using an echo‐planar imaging (EPI) sequence with the following parameters: TR/TE, 2000/30 ms; FA, 90°; 43 slices with interleaved acquisition; thickness/gap, 3.2/0 mm; FOV, 220 × 220 mm; matrix, 64 × 64; and voxel size, 3.44 × 3.44 × 3.2 mm. The participants were instructed to close their eyes, relax, remain motionless, not think of anything in particular, and not fall asleep during the resting‐state fMRI (RS‐fMRI) scanning.

### 2.6 MRI Data Processing for Defining cTBS Target Region and Detecting cTBS Modulatory Effects

A gray matter probability map in SPM 12 (threshold > 0.2) was utilized for multiple comparison corrections in all voxel‐based analyses in this study.

Group‐level preprocessing of RS‐fMRI data, aimed at comparing individuals with TS and TDC at baseline and detecting modulatory effects of cTBS in MNI space, followed these steps: (1) removal of the first 10 volumes; (2) slice timing correction; (3) head motion correction; (4) spatial normalization to standard MNI space using the EPI template; (5) regressing out the signals of WM, CSF, and the head motion parameters with the six rigid body model; (6) removal of linear trends; (7) temporal band‐pass filtering (0.01–0.1 Hz); and (8) spatial smoothing (FWHM 6 mm). Due to the non‐invasive nature of brain stimulation, achieving precision sufficient to affect only 7 voxels is not feasible. Therefore, a GPi mask was used to assess modulatory effects [22]. The mean time course of the bilateral GPi mask was extracted from the preprocessed RS‐fMRI data. Subsequently, voxel‐wise FC was calculated across the whole brain, incorporating head motion scrubbing regressors with a framewise displacement threshold of 0.2 for identifying “bad” timepoints in linear regression, addressing motion concerns [23]. The resulting correlation coefficients (*r*‐values) were transformed to *z*‐values using Fisher's *z*‐transformation.

### 2.7 Analysis I: MRI Data Processing in Original Space for Navigation Purposes

The SMA, defined in the Harvard‐Oxford cortical structural atlases with a gray matter probability threshold of 25% (http://fsl.fmrib.ox.ac.uk/fsl/fslwiki/Atlases), was selected as a mask to confine the distribution range of individual stimulation targets. The patient's T1 image was manually reoriented, followed by spatial normalization and segmentation into gray matter, white matter, and cerebrospinal fluid using SPM12 (http://www.fil.ion.ucl.ac.uk/spm/software/spm12/). The spatial normalization parameters derived from this process were subsequently applied to facilitate transformation between standard space and the original space in the subsequent fMRI data analyses.

Preprocessing of the RS‐fMRI data was performed using the DPABI toolkit (http://rfmri.org/dpabi) [24], encompassing the following steps: (1) removal of the first 10 volumes; (2) slice timing correction; (3) head motion correction; (4) co‐registration of all EPI images to the T1 image; (5) regression of signals from white matter (WM), cerebrospinal fluid (CSF), and the six rigid body head motion parameters; (6) detrending; and (7) temporal band‐pass filtering (0.01–0.1 Hz).

For the individual RS‐fMRI FC analysis of the GPi, the central voxels of the anterior third of bilateral GPis [25] in the original individual‐space images were manually selected as seeds for each participant (Figure 1A). The mean RS‐fMRI time course of these two seed ROIs, each with a radius of 4 mm (totaling seven voxels), was then extracted. Spatial smoothing (full width at half maximum—FWHM 6 mm) was applied to the preprocessed data. Subsequently, voxel‐wise FC was calculated for each participant in their original space. The voxel exhibiting the strongest FC within a depth of 4 cm beneath the scalp and within the SMA mask (AAL template) [26] was designated as the stimulation target for each participant (Figure 1A). The hemisphere with the strongest FC location determined the side for stimulation target selection (either left or right, LSMA or RSMA). The MNI coordinates of function‐specific targets in TS patients are provided in Table S1.

The CVSMA target was defined by measuring the distance from the occipital eminence to the nasal root and then moving 15% of this length forward from the Cz point of the EEG international standard lead 10–20 system (Figure 1A). The mean scalp coordinates of the CVSMA target were calculated for the *X* and *Y* axes (Table S1).

### 2.8 Stimulation Protocol

The cTBS was administered using a 70‐mm figure‐of‐8 coil equipped with a specialized air‐cooling system (Magstim Super Rapid [2], Magstim Co., Whitland, UK). Frameless stereotaxic neuronavigation (Brainsight, Rogue Research, Montreal, Canada) was employed to track the positions of both the coil and the patient's head. The resting motor threshold (RMT) was determined using the right first dorsal interosseous (FDI) muscle, targeting the left “hotspot”. The coil was positioned at a 45° angle toward the contralateral forehead, targeting the M1 area. RMT was defined as the minimum stimulator output intensity that consistently elicited a response (> 50 μV) in more than 5 out of 10 consecutive trials, plus 1 [27]. The actual stimulation intensity administered to patients averaged approximately half of the maximum stimulator output [mean of 46.19% ± 4.67% (SD)] (See Table S2 for further details).

cTBS (which uses a much shorter stimulation time than 1‐Hz rTMS and is more suitable for TS in children) has been reported to induce neurophysiological effects similar to those of inhibitory 1‐Hz rTMS [28]. Additionally, cTBS applied to the SMA in patients with chronic tic disorders has shown a significant suppressive effect on the motor network [6]. In a previous study, researchers applied 30 Hz cTBS at 90% of RMT to suppress fMRI activation during finger tapping; however, they did not observe a difference in tic severity ratings between the cTBS and sham groups. Therefore, we adopted the commonly used protocol of 50 Hz cTBS [29] at 70% of RMT.

All participants received cTBS, which consisted of three pulses per burst repeated at a rate of five bursts per second, resulting in a total of 600 pulses per train [6, 30, 31]. Three trains of cTBS were administered daily over five consecutive days. The first and second cTBS trains were spaced 15 min apart, while the second and third cTBS trains were separated by 45 min. cTBS treatment sessions were scheduled at consistent times across all five days for each patient. This stimulation protocol was selected based on prior studies [6] and our own experience, confirming the safety and efficacy of delivering 1800 pulses per day [32]. If any adverse events occur, the intervention will be promptly discontinued, and any perceived physiological or psychological discomfort in the patient will be addressed.

### 2.9 Statistical Analyses

#### 2.9.1 Clinical Assessment

All statistical analyses regarding clinical efficacy were performed using SPSS (version 25.0, IBM Corp., Armonk, NY, USA). Group differences in age and gender between TS patients and TDC were assessed using two‐sample *t*‐tests and chi‐square tests. The YGTSS scores at one week after the initiation of cTBS were compared with baseline scores using Friedman's test. Some patients underwent reassessment after two weeks of stimulation (see to Table 2), and the YGTSS scores from the three measurements (baseline, one week, and two weeks) were compared using Related‐samples Friedman's two‐way analysis of variance by ranks test.

### 2.10 Analysis II: Group Level Analysis for cTBS Modulatory Effects

#### 2.10.1 Abnormal Functional Connectivity Network in TS

To validate the findings of previous studies regarding the close relationship between the GPi and motor areas, we conducted a two‐sample *t*‐test using SPM12, incorporating head motion parameters as a covariate. This analysis aimed to explore baseline differences in GPi FC maps across the entire brain between individuals with TS and TDC. We hypothesized that abnormal FC would be evident in the SMA and that cTBS would modulate this abnormal FC toward normalcy.

### 2.11 Hypothesis I: Function‐Specific Target Offers Advantages Over Conventional Target

#### 2.11.1 Different Modulatory Effects Resulted From Different Targets

Conventional scalp‐based measurements targeting the SMA, despite aiming for the cerebral longitudinal fissure, may inadvertently stimulate either the left or right hemisphere due to inherent structural asymmetry in the human brain. This asymmetry, observed in a subset of patients, could potentially lead to clinical improvements. Given the brain's functional asymmetry, it is plausible that stimulation of the left and right SMAs may induce distinct modulatory effects. Furthermore, variation in spatial locations (as detailed in Table S1), particularly along the anterior–posterior axis due to differences in defining the target, may contribute to variations in the modulation of brain function. Therefore, a two‐way repeated measures ANOVA (with group as the between‐subjects factor and measure as the within‐subjects factor) was performed in SPM12, with head motion parameters included as a covariate, to detect differences in modulatory effects (post‐ minus pre‐cTBS) among the three targets on FC maps.

### 2.12 Hypothesis II: Functional Alteration of GPi‐Target Associated With Clinical Efficacy

Recent studies have indicated that brief, high‐dose rTMS can shorten the time required to achieve clinical efficacy. Our current research integrates cTBS as an adjunct to standard treatment protocols. We hypothesize that this supplementary intervention may accelerate the onset of clinical efficacy. This accelerated response could potentially be attributed to functional changes induced by cTBS within the “effective region” (GPi), thereby enhancing treatment outcomes. Subsequently, FC between specific targets (mean coordinates of LSMA −8, 5, 67; RSMA 6, 4, 64; radius 10 mm) and the GPis was computed. The Shapiro–Wilk test showed a significance level of *p* = 0.496 for changes in GPi‐target FC and *p* = 0.041 for the reduction in YGTSS scores. Pearson correlation analysis was then performed between the changes in GPi‐target FC and the reduction in YGTSS scores to assess whether FC correlates with the modulatory effects of cTBS.

## 3 Results

### 3.1 cTBS‐Associated Adverse Events

No cTBS‐associated adverse events, such as headache, impairment of auditory function, or epileptic seizures, were observed in any of the patients.

### 3.2 Clinical Improvement

Despite receiving stimulation for only 5 days, the YGTSS scores one week later still showed a statistically significant decrease compared to baseline (Table 2, Friedman tests are two tailed). However, the function‐specific SMA target did not demonstrate a greater reduction in YGTSS scores than the CVSMA target. YGTSS scores at the two‐week assessment were not compared due to incomplete data: 8 participants completed it in the LSMA group, only 3 in the RSMA group, and 1 in the CVSMA group.

Clinical recovery is defined as a reduction rate of ≥ 80% in YGTSS scores. Noteworthy improvement is characterized by a reduction rate of ≥ 50% and < 80%. Effectiveness is indicated by a reduction rate of ≥ 30% and < 50%. Ineffectiveness is determined when the reduction rate is less than 30%, based on clinical practice and prior research in the treatment of Tourette syndrome [33]. At the one‐week assessment, two patients in the LSMA group showed a reduction rate exceeding 30%, whereas neither the RSMA nor CVSMA groups demonstrated such changes (see Table S3).

During the one‐week assessment, five patients experienced a slight worsening of symptoms, all of whom withdrew from the study by the two‐week assessment. There was no clear evidence suggesting a relationship with cTBS treatment. These five patients were distributed as follows: two in the LSMA group, two in the RSMA group, and one in the CVSMA group. In the LSMA group, one patient exhibited a five‐point increase in the motor score, while the other exhibited a one‐point increase. In the RSMA group, one patient exhibited a one‐point increase, and the other exhibited a two‐point increase in the motor score. In the CVSMA group, both the motor score and the phonic score increased by one point each.

### 3.3 cTBS Modulatory Effects

#### 3.3.1 Abnormal Functional Connectivity Network

**FIGURE 4:**
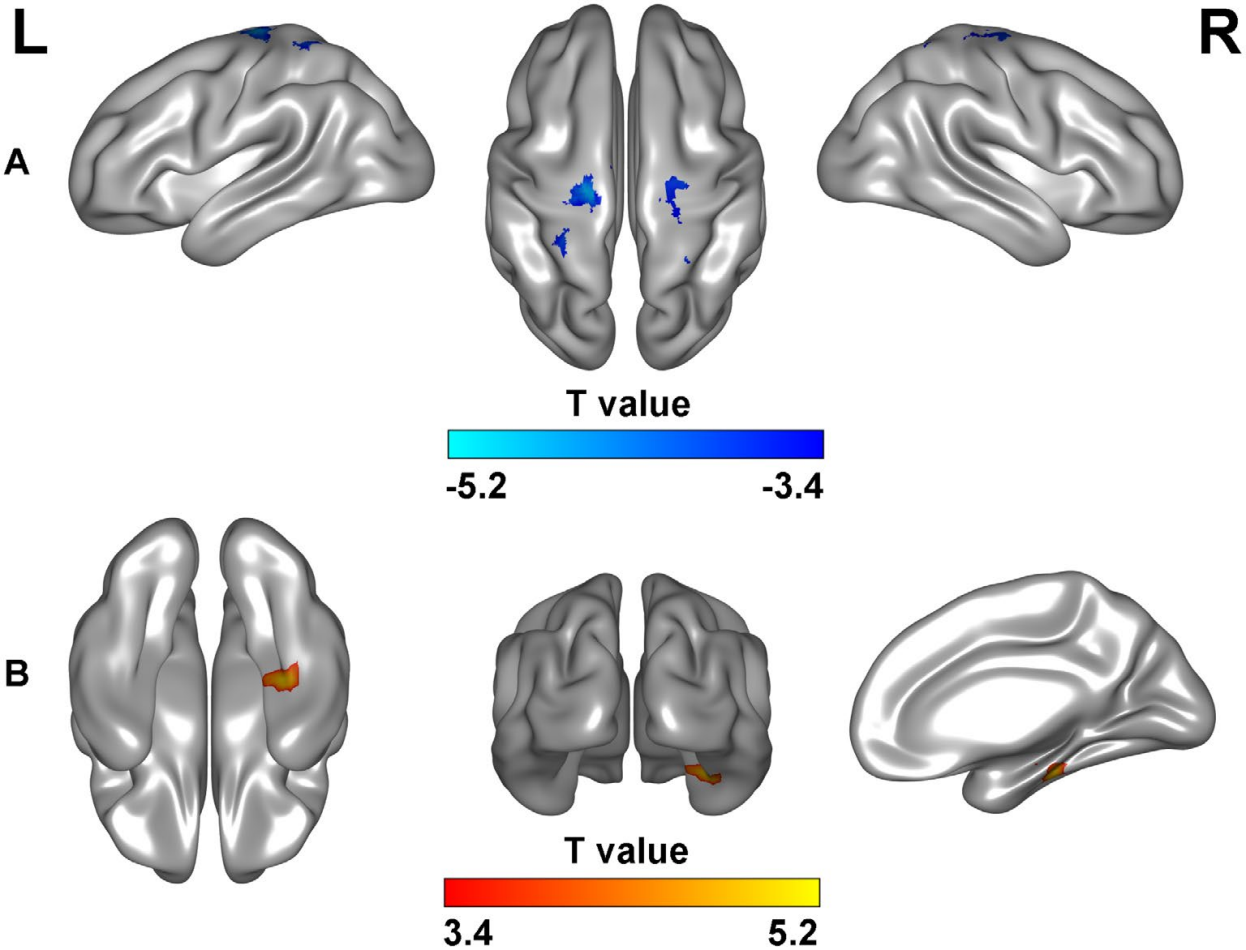
Two sample t‐tests on whole‐brain functional connectivity maps. (A) Compared to TDC at baseline, patients with Tourette's syndrome exhibited a significant reduction in functional connectivity in the left paracentral lobule/SMA (X = −15, Y = −18, Z = 63, BA4/6), and the bilateral motor cortex (X = −39, Y = −39, Z = 63, X = 30, Y = −48, Z = 69, BA6) (GRF correction: Single voxel *p* < 0.001, cluster *p* < 0.05, two tailed). (B) Following one‐week of cTBS, patients showed increased functional connectivity in the left brainstem/cerebellum and right hippocampus/Fusiform gyrus (GRF correction: Single voxel *p* < 0.001, cluster *p* < 0.05, two tailed). cTBS, Continuous theta burst stimulation; GRF, Gaussian Random Field theory; SMA, Supplementary motor area; TDC, Typical developmental children.

In comparison to TDC at baseline, individuals with TS exhibited a significant reduction in FC between the GPi and the entire brain. This reduction was particularly noticeable in the left paracentral lobule/SMA (X = −15, Y = −18, Z = 63, BA4/6), as well as the bilateral motor cortex (X = −39, Y = −39, Z = 63, X = 30, Y = −48, Z = 69, BA6) (Gaussian Random Field theory (GRF) correction: single voxel *p* < 0.001, cluster *p* < 0.05, two tailed, as shown in Figure 4A). Following one week of cTBS, the abnormally reduced FC in the motor area was replaced by increased FC in the left brainstem/cerebellum and right hippocampus/Fusiform gyrus (GRF correction: single voxel *p* < 0.001, cluster *p* < 0.05, two tailed, as shown in Figure 4B).

### 3.4 Hypothesis I: Function‐Specific Target Offers Advantages Over Conventional Target

#### 3.4.1 Different Modulatory Effects Resulted From Different Targets

**FIGURE 5:**
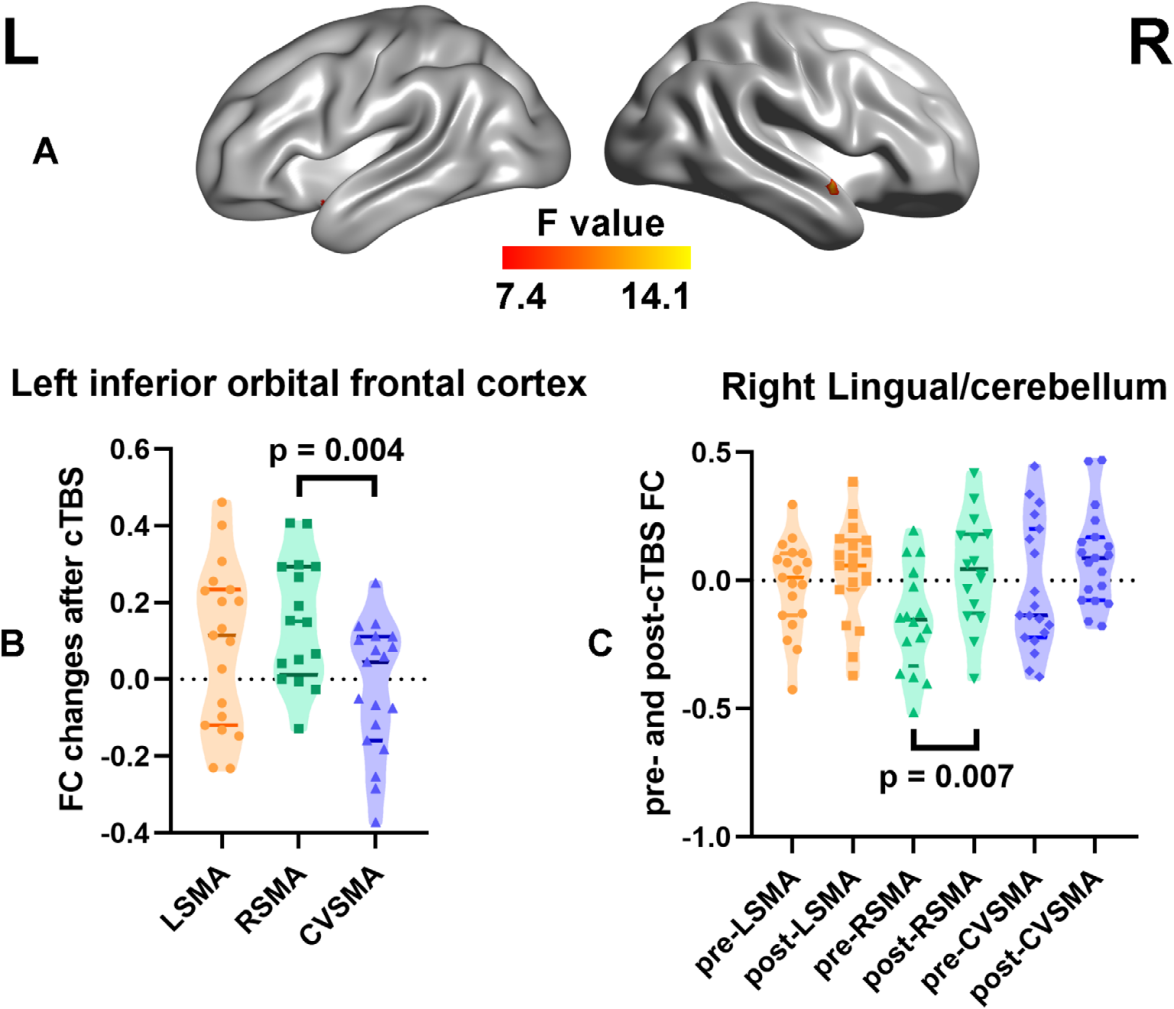
Different modulatory effects resulted from different targets. (A) A two‐way repeated measures ANOVA of the whole brain revealed significant differences among the three targets (LSMA, RSMA, and CVSMA) and measures after 5 days of stimulation (GRF correction: Single voxel *p* < 0.001, cluster *p* < 0.05). (B) Pairwise comparisons (two sample *t*‐tests) indicated that the RSMA target significantly contributed to observed differences in the left inferior orbital fontal cortex between groups. (C) Pairwise comparisons (paired *t*‐tests) indicated that the RSMA target significantly contributed to observed differences in the right lingual/cerebellum between pre‐ and post cTBS. cTBS, Continuous theta burst stimulation; CVSMA, Conventional scalp localized target of SMA; GRF, Gaussian Random Field theory; LSMA, Left SMA target; RSMA, Right SMA target; SMA, Supplementary motor area.

Significant main effects for groups were found in the left inferior orbital frontal cortex (*X* = −51, *Y* = 33, *Z* = −12, BA47), and main effects for measures were observed in the right Lingual cortex/cerebellum (X = 15, Y = −57, Z = −9, BA19) [GRF correction: single voxel *p* < 0.001, cluster *p* < 0.05, *F* (2, 51) = 7.4, one tailed, as shown in Figure 5A]. The peak zFC values of the left inferior orbital frontal cortex and the right Lingual cortex/cerebellum were extracted for pairwise comparisons, facilitating the identification of differences. These findings suggest that the RSMA target is the predominant source of observed differences on brain maps after one week cTBS (Two sample *t*‐tests, two tailed, Figure 5B; Paired *t*‐tests, two tailed, Figure 5C).

### 3.5 Hypothesis II: Functional Alteration of GPi‐Target Associated With Clinical Efficacy

**FIGURE 6:**
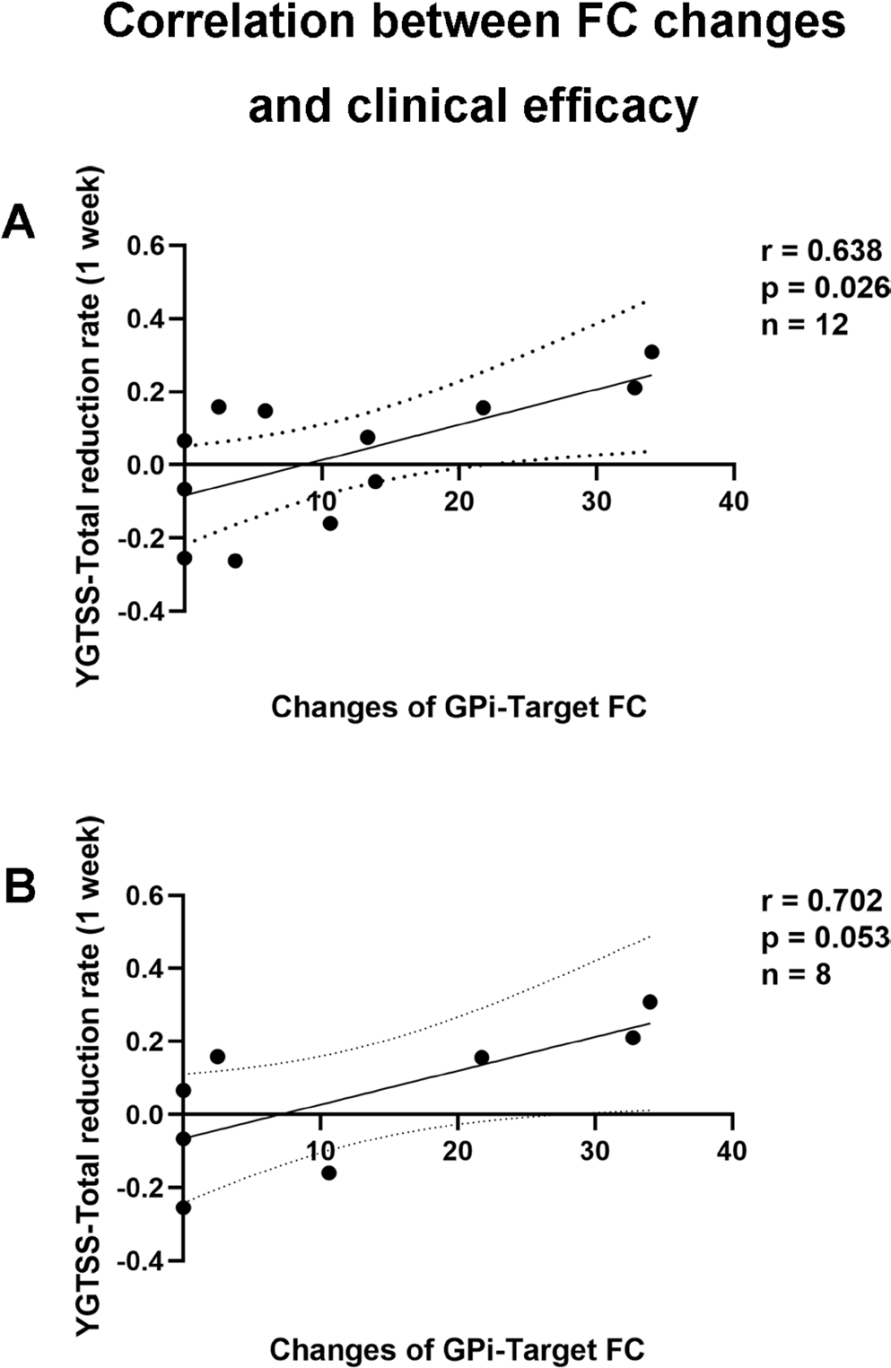
Functional alterations associated with clinical efficacy. (A) Significantly positive correlation between alterations in GPi‐target functional connectivity and reduction rate in YGTSS scores among all twelve patients evaluated at two‐week assessment. (B) Among the patients evaluated at two weeks, eight were in the left SMA target group, revealing a marginally significant positive correlation between alterations in GPi‐target functional connectivity and reduction rate in YGTSS scores. GPi, Globus pallidus internus; LSMA, Left SMA target; SMA, Supplementary motor areas; YGTSS, Yale Global Tic Severity Scale.

Twelve patients completed the two‐week assessment. They all come from a homogeneous population, i.e., Tourette Syndrome patients who received cTBS intervention with identical stimulation parameters targeting the same brain region (SMA). The only difference lay in the method of target localization, i.e., traditional versus function‐specific. Both LSMA and RSMA are function‐specific targets, distinguished by whether the peak voxel of FC in the GPi‐SMA falls on the left or right side. Therefore, we pooled the data from the different targets for analysis. Since only one patient belonged to the CVSMA group, further analysis focused exclusively on the function‐specific target groups. A significantly positive correlation was observed between changes in GPi‐target FC and reductions in YGTSS scores after one‐week cTBS (*r* = 0.638, 95% confidence interval 0.102 to 0.887, *p* = 0.026, *n* = 12, two tailed, Figure 6A). This significant correlation, primarily observed in the LSMA group (*r* = 0.702, 95% confidence interval −0.115 to 0.941, *p* = 0.053, *n* = 8, two tailed, Figure 6B), suggests that changes in GPi‐target FC predominantly influenced YGTSS score reductions.

All brain function results were visualized using BrainNet Viewer (http://www.nitrc.org/projects/bnv/) [34].

## 4 Discussion

In the current study, we defined function‐specific individualized targets for the precise treatment of TS using cTBS. Although the function‐specific SMA targets did not show a significant difference in reducing tic severity compared to CVSMA at the one‐week assessment, we observed that stimulation of the left SMA target led to a YGTSS reduction rate exceeding 30%. cTBS applied to the left SMA target significantly altered the functional connectivity between the GPi and cortical regions, specifically the left inferior orbital frontal cortex and right Lingual cortex/cerebellum. Furthermore, changes in functional connectivity between the GPi and the function‐specific SMA target were significantly associated with the reduction in the YGTSS total score after one week. Previous studies have reported a reduction in tic severity after six months of stimulation targeting a conventional SMA target [12, 35], whereas we observed alterations in brain function after only five days of stimulation, with clinical efficacy becoming evident within just two weeks.

### 4.1 Clinical Efficacy Defined by YGTSS

The primary outcome is clinical efficacy, as defined by the YGTSS [36, 37]. The YGTSS assesses the severity of motor and phonic tics experienced in the previous week (score range: 0–50) and evaluates their impact on quality of life (score range: 0–50) in both children and adults. It is commonly utilized to evaluate treatment efficacy following a minimum of four weeks of therapy. Baseline inclusion criteria and effectiveness standards vary across studies. In this study, we consistently applied diagnostic criteria and clinical efficacy assessment standards used in our department (details are provided in the Results section). Several studies have documented rapid therapeutic effects through high‐density stimulation over short durations [7, 8, 9, 10]. Given the experimental nature of rTMS therapy for TS, we integrated supplementary stimulation alongside conventional medication with the aim of shortening treatment duration while maintaining therapeutic effectiveness.

### 4.2 Defining Stimulation Target in Superficial Cortex

The GPi is frequently targeted in studies for treating TS [38]. A randomized double‐blind sham stimulation‐controlled trial demonstrated a significant reduction in tics with GPi targeting [39]. Tics can arise from basal ganglia abnormalities [40] and dysfunctions in GABAergic networks [41]. DBS studies have shown that during tic events, individual neurons in the external and internal segments of the GP exhibit transient, tic‐related changes in firing rates. This finding suggests that basal ganglia regulation is a key mechanism underlying the expression of individual tics [42]. Patients with TS exhibited decreased GABAA receptor binding in the bilateral GP, while increased binding occurs in the posterior cingulate cortex and cerebellum [43]. An fMRI study suggests increased excitatory neurotransmission and enhanced connectivity within basal ganglia–thalamo–cortical circuits [44]. Task activation‐guided cTBS targeting the SMA significantly reduced cortical brain activation in chronic tic disorder patients after 2 days of stimulation compared to sham cTBS [6]. However, neurophysiological recordings from deep brain structures suggest that tics originate primarily from GP and thalamus neuronal dysfunction, rather than cortical dysfunction [45]. In our current study, we initially validate the functional relationship between the GPi and the motor area. Consistent with prior research, our findings support the selection of SMA as a stimulation target (Figure 4A) [6]. Nevertheless, the SMA spans approximately 6 cm in length and 4 cm in width [26], posing challenges in pinpointing the exact stimulation site within it. This study holds practical significance for TS treatment, highlighting the need for further clarification on selecting the SMA as the target region and specifying the stimulation site within it.

### 4.3 Spatial Location of Targets and Its Modulatory Effects

The mean coordinates of the LSMA were −8, 5, 67, and for the RSMA, they were 6, 4, 64 (please refer to the Table S1 for details). The mean coordinates of the CVSMA were 0 and 35 for the *X* and *Y* axes, respectively. The CVSMA and the function‐specific target exhibited substantial spatial discrepancies, particularly along the anterior–posterior axis of the brain. Decades ago, researchers divided the SMA into functional segments, labeling the portion anterior to the Y axis 20 as pre‐SMA [46]. More recently, a delineation method using a line crossing the anterior commissure has been adopted, designating the posterior section as posterior SMA or SMA‐proper [47, 48, 49]. The pre‐SMA is predominantly involved in cognitive planning processes, whereas SMA‐proper is more closely linked to motor execution [50, 51, 52]. The function‐specific targets in the current study were all located in the SMA‐proper, while the CVSMA targets were in the pre‐SMA portion. This difference could be attributed to the potentially superior modulatory effects of function‐specific targets. Although previous studies concluded that cTBS to the motor area induced a similar inhibitory effect to 1‐Hz rTMS, this finding was only based on the measurement of the motor‐evoked potential (MEP) [29], which reflects cortical excitability. The facilitatory effect on functional connectivity indicated by the blood‐oxygen‐level‐dependent (BOLD) signal might represent an improvement in brain function; TS patients may be able to suppress their involuntary movements via enhanced motor‐related cognitive control.

Previous research has identified abnormal structure in the orbital frontal cortex (OFC) in TS [53]. Animal studies using single‐neuron recordings have shown that functional changes in coherence across cortico‐striato‐thalamic circuits (including the OFC) are associated with hyperactivity and repetitive behaviors [54]. The OFC and dorsal striatum play a key role in inhibitory control, with increased OFC input into the dorsal striatum possibly indicating enhanced cognitive control [55]. Our findings demonstrate that targeting the right SMA induces the greatest FC change in the OFC between targets (Figure 5B), suggesting a potential enhancement in the modulatory function of the OFC, although no correlation with the severity of tics was observed. Additionally, we observed an increase in functional connectivity in the Lingual/cerebellum following cTBS applied to the right SMA target (Figure 5C). The OFC, precentral gyrus, and cerebellum are part of the fronto‐cerebellar circuits that serve cognitive and executive functions, specifically related to motor operations and the control of eye movements [56]. cTBS may induce changes in functional connectivity within these motor‐related networks, potentially modifying motor control functions in individuals with TS. Our findings suggest that targeting function‐specific SMA regions may facilitate improved functioning of motor‐related networks and further enhance motor control.

### 4.4 Alteration of GPi‐Target Functional Connectivity Associates With Clinical Efficacy

YGTSS assessments are typically conducted four weeks after treatment initiation in medication research studies [36]. Following the precedent of rapid efficacy observed in short‐term, high‐dose rTMS studies, assessments in our current study were performed at one‐ and two‐week post cTBS. The one‐week assessment did not reveal group‐level differences; however, the left SMA target demonstrated an advantage (Table S3). Twelve TS patients completed the assessment after two weeks, revealing that changes in GPi‐target FC were significantly positively correlated with reductions in YGTSS scores (Figure 6A). Further analysis indicated that this correlation was predominantly driven by the left SMA target (Figure 6B). Larger FC changes after cTBS were associated with improved clinical outcomes. This finding is consistent with our baseline comparison, where functional connectivity between the GPi and SMA in TS patients was lower compared to TDC (Figure 4A). Applying cTBS to the function‐specific SMA target indirectly modulated GPi function, with enhanced functional connectivity between the target and GPi reflecting improved GPi function. This speculation aligns with previous DBS studies suggesting bilateral GPi modulation as a treatment for TS [57, 58], although benefits from function‐specific targets did not reach statistically significant levels. We avoided using a short‐term, high‐dose stimulation protocol, which, while possibly effective, lacks practical relevance in clinical settings. However, the changes in GPi‐target FC associated with clinical efficacy after 5 days of stimulation suggest the potential for function‐specific targeted treatment of TS patients, thereby minimizing treatment costs for individuals. Future research should further investigate the duration of single sessions and the intervals between sessions for patients responding to cTBS.

## 5 Limitations

This study had several limitations. First, the sample size was small. Although the sample size was constrained by the number of participants meeting our strict inclusion criteria, we acknowledge that a larger sample size would enhance statistical power and improve the generalizability of our findings. For future research, we recommend recruiting larger and more diverse cohorts to validate our findings and explore potential subgroup effects. Furthermore, we suggest leveraging multi‐center collaborations to increase sample sizes and improve the representativeness of the study population. Despite the small sample size in this study, the current results remain robust. We conducted rigorous statistical analyses and, where necessary, applied appropriate methods (e.g., the non‐parametric Friedman test) to ensure the reliability of our conclusions. Second, the seed time series for FC were extracted from bilateral GPi, and the ROIs were small. This may have led to weak FC intensity, potentially affecting target definition. Third, 67% of the patients who completed the two‐week assessment belonged to the left SMA group, suggesting a potentially greater clinical impact from the left SMA. However, it is also plausible that patients in the right SMA group and the CVSMA group dropped out due to a perceived lack of efficacy. Fourth, the stimulation duration was limited to 5 days, which may be considered rather short for TS treatment. A one‐month stimulation duration is acceptable in China, as it aligns with the school holiday period. Future studies could extend the stimulation duration to one month. Fifth, the follow‐up period of 2 weeks was short. While the 2‐week follow‐up allowed us to capture short‐term clinical improvements, we acknowledge that this timeframe does not provide sufficient insights into the long‐term efficacy of cTBS treatment. Future research should incorporate follow‐up assessments at 6 months or 1 year post‐treatment to better understand the long‐term efficacy and potential durability of the observed clinical improvements.

## 6 Conclusions

The current study explored a new target for cTBS based on previous findings from DBS and rTMS studies. Five days of cTBS significantly reduced YGTSS scores. Function‐specific SMA targets demonstrated better clinical efficacy than CVSMA targets. Alterations in GPi‐target FC were associated with clinical efficacy, with larger FC changes after cTBS leading to improved clinical outcomes. The enhanced FC between the target and GPi indirectly reflected improved GPi function, suggesting that modulating bilateral GPi function could effectively treat TS. The function‐specific location in SMA emerges as a promising target for cTBS treatment of TS and warrants further investigation. These results are preliminary and highlight the need for long‐term studies to establish the clinical relevance of cTBS treatment for Tourette's syndrome.

## Author Contributions

J.W., Y.F.Z., and J.H.F. contributed to the conception and design of the study; Y.W., X.L.L., X.P.D., Y.T.L., X.Q.C., and Q.Y.S. contributed to the acquisition; J.W. and J.Y. contributed to the analysis of data, drafting the text, and preparing the figures.

## Consent

Informed consent forms have been given to participants and their parents.

## Conflicts of Interest

The authors declare no conflicts of interest.

## Supporting information


**Tables S1–S3**:

## Data Availability

Due to the clinical nature of the data, the findings of this study are supported by data that is not freely available. These data can be made accessible by the corresponding author upon reasonable request. However, a formal data‐sharing agreement must be established before any data can be shared.
