# Supplementary material for: Function‐Specific Localization in the Supplementary Motor Area: A Potential Effective Target for Tourette Syndrome
Source: CNS Neurosci Ther. 2025 Feb 21;31(2):e70280. doi: 10.1111/cns.70280 (PMC11843473; doi:10.1111/cns.70280)
Supplement: Supplementary file 1 — Tables S1–S3: [file CNS-31-e70280-s001.docx]

**Supplementary Materials**

**Function-Specific Localization in the Supplementary Motor Area: A Potential Effective Target for Tourette Syndrome**

Jue Wang^1†^, Juan Yue^2†^, Ye Wang^3†^, Xiao-Long Li^4^, Xin-Ping Deng^4^, Yu-Ting Lou^3^, Liu-Yan Gao^3^, Xiao-Quan Chen^3^, Qun-Yan Su^5^, Yu-Feng Zang^4^, Jian-Hua Feng^3*^

Running title: Function-specific target for TS

1 Institute of sports medicine and health, Chengdu Sport University, Chengdu 641418, China

2 TMS Center, Hangzhou Normal University Affiliated Deqing Hospital, Huzhou, 313201, China

3 Department of Pediatrics, the Second Affiliated Hospital, Zhejiang University School of Medicine, Hangzhou 310058, China

4 Center for Cognition and Brain Disorders, The Affiliated Hospital of Hangzhou Normal University, Hangzhou 310015, China

5 Department of Pediatrics, Taizhou Woman and Children’s Hospital, Taizhou 318000, China

Correspondence to: Jian-Hua Feng, Zijingang Campus of Zhejiang University, 866 Yuhangtang Road, Xihu District, Hangzhou 310058, China.

E-mail: [hzhz87083886@zju.edu.cn](mailto:hzhz87083886@zju.edu.cn).

**^†^These authors contributed equally to this work.**

**Table 1. The MNI coordinates of stimulation targets**

| Group | SubID | Stimulation target (MNI coordinate) | | |
| --- | --- | --- | --- | --- |
|  |  | X | Y | Z |
| LSMA | Sub001 | -7 | 18 | 69 |
|  | Sub002 | -15 | 15 | 68 |
|  | Sub003 | -5 | 21 | 60 |
|  | Sub004 | -11 | 24 | 65 |
|  | Sub005 | -3 | 6 | 60 |
|  | Sub006 | -3 | 0 | 69 |
|  | Sub007 | -9 | 3 | 72 |
|  | Sub008 | -6 | 3 | 63 |
|  | Sub009 | -6 | -12 | 66 |
|  | Sub010 | -3 | 3 | 75 |
|  | Sub011 | -12 | 6 | 69 |
|  | Sub012 | -3 | -3 | 66 |
|  | Sub013 | -3 | 12 | 63 |
|  | Sub014 | -9 | -6 | 78 |
|  | Sub015 | -9 | 6 | 75 |
|  | Sub016 | -6 | 12 | 57 |
|  | Sub017 | -6 | -6 | 66 |
|  | Sub018 | -9 | -6 | 63 |
|  | Sub019 | -18 | 0 | 63 |
|  | Mean coordinate | -8 | 5 | 67 |
| RSMA | Sub020 | 15 | 0 | 69 |
|  | Sub021 | 3 | 0 | 63 |
|  | Sub022 | 6 | -21 | 72 |
|  | Sub023 | 9 | -9 | 63 |
|  | Sub024 | 3 | 24 | 54 |
|  | Sub025 | 12 | -6 | 66 |
|  | Sub026 | 12 | 21 | 57 |
|  | Sub027 | 3 | 6 | 66 |
|  | Sub028 | 3 | 6 | 66 |
|  | Sub029 | 6 | 3 | 60 |
|  | Sub030 | 3 | 6 | 60 |
|  | Sub031 | 9 | 3 | 66 |
|  | Sub032 | 3 | -9 | 75 |
|  | Sub033 | 9 | 24 | 60 |
|  | Sub034 | 9 | 3 | 66 |
|  | Sub035 | 3 | 15 | 57 |
|  | Mean coordinate | 6 | 4 | 64 |
| CVSMA | Mean coordinate | 0 | 35 |  |

MNI: Montreal Neurological Institute; SMA: supplementary motor area; LSMA: left SMA target; RSMA: right SMA target; CVSMA: conventional scalp-localized SMA target.

**Table 2. The stimulation intensity for Tourette's syndrome patient**

| Group | Sub ID | RMT | Output intensity corresponding to 70% RMT |
| --- | --- | --- | --- |
| LSMA | Sub001 | 60 | 42 |
|  | Sub002 | 70 | 50 |
|  | Sub003 | 66 | 46 |
|  | Sub004 | 70 | 50 |
|  | Sub005 | 65 | 46 |
|  | Sub006 | 65 | 46 |
|  | Sub007 | 45 | 32 |
|  | Sub008 | 70 | 50 |
|  | Sub009 | 57 | 40 |
|  | Sub010 | 70 | 50 |
|  | Sub011 | 70 | 50 |
|  | Sub012 | 70 | 49 |
|  | Sub013 | 70 | 50 |
|  | Sub014 | 69 | 48 |
|  | Sub015 | 67 | 47 |
|  | Sub016 | 70 | 50 |
|  | Sub017 | 59 | 41 |
|  | Sub018 | 69 | 48 |
|  | Sub019 | 69 | 48 |
| RSMA | Sub020 | 70 | 50 |
|  | Sub021 | 60 | 42 |
|  | Sub022 | 70 | 50 |
|  | Sub023 | 70 | 50 |
|  | Sub024 | 70 | 50 |
|  | Sub025 | 58 | 41 |
|  | Sub026 | 70 | 50 |
|  | Sub027 | 57 | 40 |
|  | Sub028 | 70 | 50 |
|  | Sub029 | 60 | 42 |
|  | Sub030 | 70 | 49 |
|  | Sub031 | 67 | 47 |
|  | Sub032 | 67 | 47 |
|  | Sub033 | 70 | 50 |
|  | Sub034 | 64 | 45 |
|  | Sub035 | 70 | 49 |
| CVSMA | Sub036 | 70 | 50 |
|  | Sub037 | 70 | 50 |
|  | Sub038 | 48 | 34 |
|  | Sub039 | 58 | 40 |
|  | Sub040 | 53 | 37 |
|  | Sub041 | 66 | 46 |
|  | Sub042 | 66 | 46 |
|  | Sub043 | 66 | 46 |
|  | Sub044 | 60 | 42 |
|  | Sub045 | 68 | 48 |
|  | Sub046 | 70 | 50 |
|  | Sub047 | 70 | 50 |
|  | Sub048 | 54 | 38 |
|  | Sub049 | 70 | 50 |
|  | Sub050 | 70 | 50 |
|  | Sub051 | 57 | 40 |
|  | Sub052 | 60 | 42 |
|  | Sub053 | 70 | 50 |
|  | Sub054 | 70 | 50 |
|  | Mean ± SD | 65.37 ± 6.29 | 46.19 ± 4.67 |

RMT: resting motor threshold; SMA: supplementary motor area; LSMA: left SMA target; RSMA: right SMA target; CVSMA: conventional scalp-localized SMA target.

**Table 3 YGTSS score reduction rate for TS patients**

| Groups | 1-week YGTSS reduction rate (*n/N*) | | | 2-week YGTSS reduction rate (*n/N*) | | | |
| --- | --- | --- | --- | --- | --- | --- | --- |
|  | Effectiveness | Ineffectiveness | Worsen | Noteworthy improvement | Effectiveness | Ineffectiveness | Worsen |
| LSMA | 34%, 33%, 33%  (3/19) | 11/19 | -6%, -7% (2/19) | 64%, 56%, 70%  (3/8) | 41%  (1/8) | (4/8) | -- |
| RSMA | -- | 15/16 | -8%, -5% (2/16) | 56% (1/3) | -- | (2/3) | -- |
| CVSMA | -- | 18/19 | -3% (1/19) | -- |  | (1/1) | -- |

YGTSS: Yale Global Tic Severity Scale; Effectiveness: reduction rate of ≥ 30% and < 50%; Ineffectiveness: reduction rate < 30%; Worsen: reduction rate < 0; Noteworthy improvement: reduction rate of ≥ 50% and < 80%; SMA: supplementary motor area; LSMA: left SMA target; RSMA: right SMA target; CVSMA: conventional scalp-localized SMA target.
